# History of suicidal behavior and clozapine prescribing among people with schizophrenia in China: a cohort study

**DOI:** 10.1186/s12888-024-05893-y

**Published:** 2024-06-12

**Authors:** Yi Yin, Chen Lin, Lijing Wei, Jinghui Tong, Junchao Huang, Baopeng Tian, Shuping Tan, Zhiren Wang, Fude Yang, Yongsheng Tong, Song Chen, L. Elliot Hong, Yunlong Tan

**Affiliations:** 1 Peking University HuiLongGuan Clinical Medical School, Beijing HuiLongGuan Hospital, Beijing, People’s Republic of China; 2 Beijing Suicide Research and Prevention Center, World Health Organization Collaborating Center for Research and Training in Suicide Prevention, Beijing, People’s Republic of China; 3 Maryland Psychiatric Research Center, Department of Psychiatry, University of Maryland School of Medicine, Baltimore, United States of America

**Keywords:** Schizophrenia, Suicide, Clozapine, Antipsychotic, Self-harm

## Abstract

**Background:**

Clozapine is an off-label drug used in most countries to prevent suicide in individuals with schizophrenia. However, few studies have reported real-world prescription practices. This study aimed to explore the association between a history of suicidal behavior and clozapine prescribing during eight weeks of hospitalization for individuals with early-stage schizophrenia.

**Methods:**

This observational cohort study used routine health data collected from a mental health hospital in Beijing, China. The study included 1057 inpatients who had schizophrenia onset within 3 years. History of suicidal behavior was coded from reviewing medical notes according to the Columbia Suicide Severity Rating Scale. Information on antipsychotic use during hospitalization was extracted from the prescription records. Time to clozapine use was analyzed using Cox regression models adjusted for sociodemographic and clinical covariates.

**Results:**

The prevalence rates of self-harm, suicidal behavior, and suicide attempt were 12.3%, 7.5%, and 5.4%, respectively. A history of self-harm history was positively associated with clozapine uses upon admission (4.1% vs. 0.8%, exact *p* = 0.009). Among those who had not used clozapine and had no clozapine contraindication, A history of suicidal behavior increased the possibility of switch to clozapine within 56 days after admission (Hazard Ratio[95% CI], 6.09[2.08–17.83]) or during hospitalization (4.18[1.62–10.78]).

**Conclusion:**

The use of clozapine for early-stage schizophrenia was more frequent among those with suicidal behavior than among those without suicidal behavior in China, although the drug instructions do not label its use for suicide risk.

**Supplementary Information:**

The online version contains supplementary material available at 10.1186/s12888-024-05893-y.

## Background

Globally, 4–20% of individuals with schizophrenia die by suicide [1, 2]. The suicide rate among individuals with schizophrenia peaks in an early stage. The risk of suicide increases by 80% after a diagnosis of schizophrenia [3]. In a study from western China, suicide was estimated to cause the largest number of potential years of life lost (31.2 years) among all causes of mortality [4].

A history of self-harm is a significant risk factor for suicide. Approximately one quarter of the individuals with schizophrenia attempt suicide [5]. This prevalence does not differ between age subgroups or sexes, although onset before age 25 years is associated with higher suicide-attempt history (41.8%) [5].

Drug therapy is the first-line treatment for schizophrenia. Previous research has provided evidence that clozapine use has an extra benefit of decreasing suicide risk compared to other antipsychotics [6]. The risk of subsequent suicide attempts and suicides decreased by 36% in a Finnish cohort and 34% in a Swedish cohort [7]. In Danish outpatients with treatment-resistant schizophrenia, the risk of self-harm was higher for non-clozapine antipsychotics than clozapine (hazard ratio [HR]: 1.36) [8]. Moreover, clozapine has shown the strongest protective effect against hospitalization for self-harm compared to other second-generation antipsychotics for individuals with initial diagnoses of schizophrenic disorders in Taiwan [9]. Continuous treatment with clozapine is associated with a 44% lower long-term mortality rate than treatment with other antipsychotics [10].

However, only the United States has approved the use of clozapine when individuals with schizophrenia have recurrent suicide risk [11]. The American Psychiatric Association [12] recommends clozapine as a treatment for individuals with schizophrenia and substantial suicide risk after using other treatments (Level 2B evidence). In China’s first national practice guideline for treating mental disorders [13], schizophrenia with suicide attempts is an indication of using electric convulsive treatment (ECT) rather than clozapine. In China, the clozapine label warns that it should only be prescribed for persistent symptoms after adequate treatment with two antipsychotics [14]. However, according to the Chinese Medical Association’s second guidelines on the prevention and treatment of schizophrenia, one indication for clozapine use is schizophrenia with serious suicide risk [15] (Details in supplementary materials).

Clozapine underutilization is widespread worldwide [16, 17]. Clozapine use can cause rare but serious side effects including severe neutropenia [11]. Thus, some psychiatrists have “clozaphobia” and are fearful of prescribing clozapine [18]. However, a meta-analysis of randomized controlled trials and cohort studies did not support the increased rates of neutropenia after clozapine use [19]. A recent study showed that the risk of clozapine-induced agranulocytosis decreases steeply over time, and fatality in patients with agranulocytosis is very low [20]. Moreover, studies on benign (ethnic) neutropenia suggest that North European and Scandinavian individuals are much more sensitive to severe neutropenia secondary to clozapine than people with other genetic background. However, individuals with benign ethnic neutropenia do not have increased infection or impaired phagocytosis compared to the general population [21]. At least seven countries have modified clozapine monitoring criteria for patients diagnosed with benign ethnic neutropenia [22]. The Food and Drug Administration of United States also relaxed the “no blood, no drug” regulations, which is largest perceived barrier to increasing clozapine utilization [23].

In clinical practice, psychiatrists prescribe clozapine to patients with schizophrenia who fail to respond adequately. A survey found that approximately 31.9% of Chinese patients with schizophrenia received clozapine, which was higher than the average prevalence (18.4%) in Asia [24]. However, to date, no study has compared the prescription patterns among patients with schizophrenia with or without a history of suicidal behavior.

We conducted a real-world, retrospective, cohort study among individuals with early-stage schizophrenia. This study aimed to determine whether a lifetime history of suicidal behavior was significantly associated with a higher possibility of switching prescriptions to clozapine during hospitalization. These findings presented the treatment for schizophrenia with suicide risk in a large developing country.

## Methods

This was an observational cohort study.

### Data source

Data were obtained from routine electronic medical records (EMR) at Beijing HuiLongGuan Hospital. This psychiatric hospital, with approximately 1,300 beds, is the largest psychiatric hospital in Beijing. In China, psychiatric hospitals mainly provide mental health care for patients with both acute-phase and chronic-phase schizophrenia. However, community-based care is limited.

The database comprised demographics, diagnoses, medical notes, and medication prescription claims.

### Participants

Inclusion criteria were: (a) Chinese citizen; (b) hospitalized from February 2, 2015 (the date start of this EMR system) to December 31, 2020; (c) diagnosis of schizophrenia (International Classification of Diseases Tenth Revision [ICD-10]: F20) at discharge; (d) schizophrenia onset within 36 months, defined “the early stage”;and (e) age < 60 years. Schizophrenia onset was defined as the first manifested of psychotic symptoms (delusions, hallucinations, and disorganized behavior) [25]. Psychiatrists obtained onset information from the patients and family caregivers.

The exclusion criteria were: (a) age at schizophrenia onset > 60 years old (due to higher possibility of misclassification); (b) history of hospitalization with schizophrenia in the EMR; (c) without medical notes; and (d) without any antipsychotic prescriptions.

### Measurements

*Demographics*. Data on sex (female/male) and age at admission were obtained. Age at admission (years) was stratified into three groups (10–24/25–39/40–59 years).

*Self-harm history*. We manually reviewed free-text clinical notes and coded the presence of lifetime suicide attempts, aborted attempts, interrupted attempts, preparatory acts, and non-suicidal self-injury (NSSI) according to the Columbia-Suicide Severity Rating Scale [26]. In addition, we coded another subtype for self-harm as psychotic-symptom-related self-harm. For example, a patient cuts the skin of his head because she/he believes an intelligence agency has embedded a chip into her/his head.

*Antipsychotic use.* We extracted all prescriptions records of the patients from the EMR including the generic drug name, prescription start date, and prescription end date during hospitalization. We used the Anatomical Therapeutic Chemical Classification System to identify antipsychotics (N05A), except lithium (N05AN01).

*Treatment history*. The definition for minimally-treated patients comprised those who did not meet any of the following criteria before being admitted to the inpatient department: (a) had continuous, sufficient dosage of antipsychotic drugs ≥ 14 days; (b) had previous hospital stays in any psychiatric hospital for psychotic symptoms treatment ≥ 14 days; and (c) used ECT within 3 months.

*Lab tests*. Information on the white blood cell count (WBC) and absolute neutrophil count (ANC) on admission was extracted. Clozapine contraindications were defined as WBC < 3500/ mm³: or ANC < 2000/ mm³ [14].

*Chronic comorbid physical diseases.* We used the Charlson Comorbidity Index [27] and the ICD-10 coding algorithm was developed by Quan [28].

*Follow-up*. The index date was the date for the first admission. An outcome event was defined as the days of hospital stay until the patient first received clozapine. Follow-up was censored on the discharge date or death date or December 31, 2020 (the day the study ended).

### Statistical analyses

We tested group differences for demographic and clinical characteristics using *χ*^2^ or Fisher’s exact tests for categorical variables. For variables that violated normality, we performed the Mann-Whitney U rank-sum test to determine the trends between the two groups. We employed a Cox proportional hazards (PH) model to explore whether patients with a history of self-harm had a higher risk of changing their prescription to clozapine.

Python 3 was used for cleaning data. Statistical analyses were performed in Stata 15.0 for Windows (Stata Corporation, College Station, Texas, the United States). Significance level was set at two-side *p*-value < 0.05.

### Ethics

This study was approved by the Institutional Review Boards of the Beijing HuiLongGuan Hospital, Beijing, China.

The institutional Review Boards of the Beijing HuiLongGuan Hospital waived the requirement for informed consent from the participants because the information was obtained from routine medical records according to local regulations. Beijing HuiLongGuan Hospital approved the use of routine electronic medical records data.

We anonymized the data and held them in a safe setting.

## Results

### Demographics and clinical characteristics

**Fig. 1:**
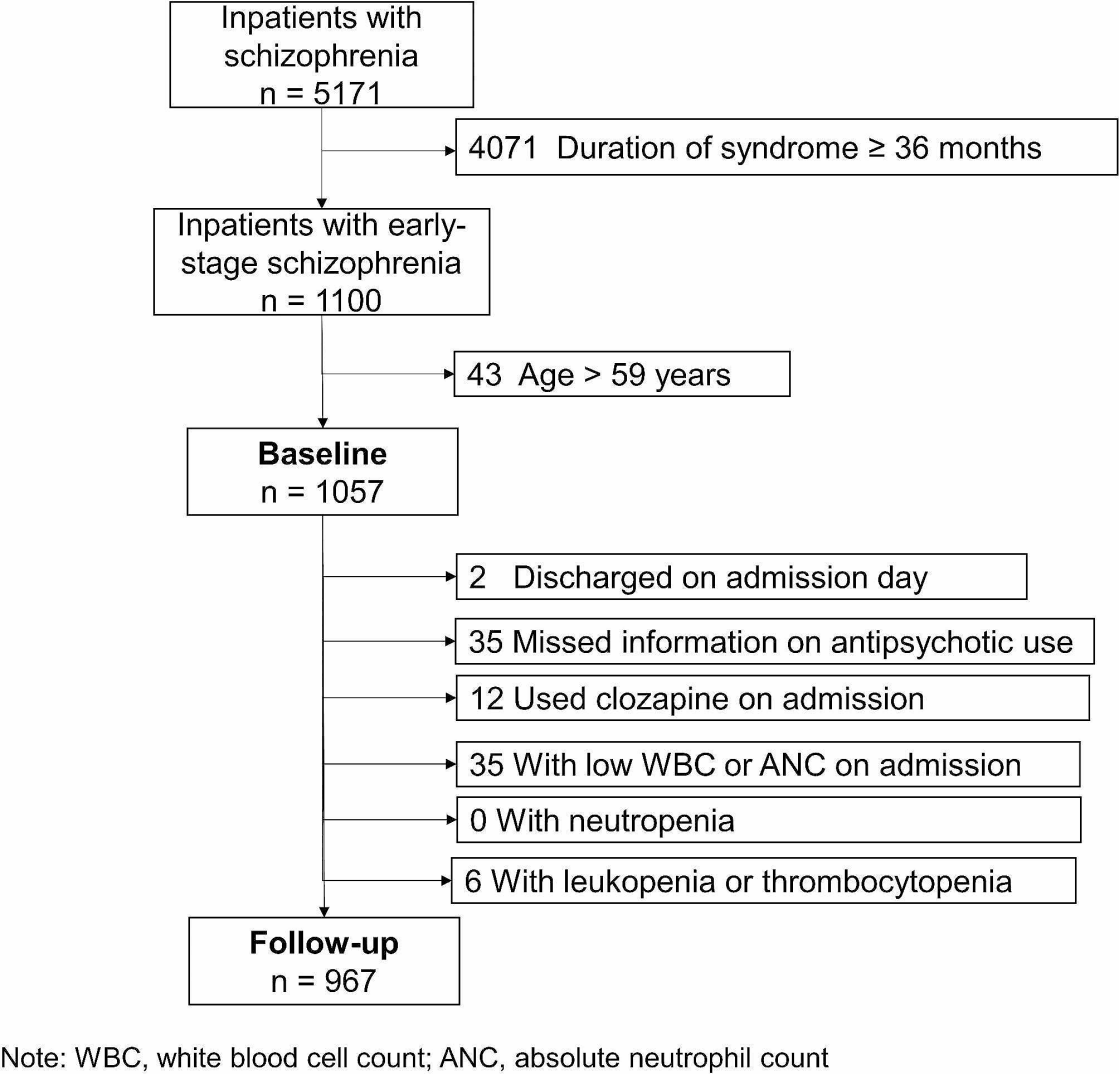
Flowchart of individuals with schizophrenia at baseline and follow-up

The study included 1057 patients in the baseline analysis (Table 1; Fig. 1). The median patient age was 26 (interquartile range [IQR] = 15; range: 13–59) years; approximately half (55.1%) were females. The mean duration of schizophrenia was 0.9 (Standard Deviation [SD] = 0.8) years.

**Table 1:** Characteristics of individuals with early-phase schizophrenia at baseline (*N* = 1057)

Variables	*n*	(%)
Sex		
Male	475	44.9%
Female	582	55.1%
Age groups		
10–24	461	43.6%
25–39	413	39.1%
40–59	183	17.3%
Treatment history		
Treated	75	7.1%
Minimal treated	982	92.9%
WBC < 3500/ mm³:	13	1.5%
ANC < 2000/ mm³:	34	3.9%
Clozapine contraindication a	41	3.9%
Charlson comorbidity index		
0–1	960	90.8%
2–3	86	8.1%
>3	11	1.0%

*Abbreviations*: WBC, white blood cell count; ANC, absolute neutrophil count; N, number.

Note: a. Clozapine contraindication was defined as WBC < 3500/ mm³, ANC < 2000/ mm³, or with a diagnosis of leukopenia or thrombocytopenia.

### Prevalence of each type of self-harm

Approximately 12.3% of patients had a history of self-harm. The prevalence of suicidal behavior, suicide attempt, interrupted attempt, and aborted attempt, NSSI, SHPS, preparatory acts was 7.5%, 5.4%, 1.8%, 0.8%, 4.7%, 2.4%, and 0.3%, respectively. The prevalence of NSSI (7.2%) was higher among those aged between 10 and 24 than that in other age groups (25–39, 3.2%; 40–59, 2.2%; *χ*^2^ = 10.95, *p* = 0.004). The prevalence of other types of self-harm did not differ between the age groups. Moreover, no difference in self-harm history was found between the sexes. Since our data were extracted from the free text of medical records, only 52 of 130 participants with a history of self-harm had records for their date of self-harm. Twenty-three patients had a history of self-harm within 30 days before hospitalization, and 15 harmed themselves within 7 days before hospitalization (Supplementary Table S1).

### Antipsychotic use on admission by each type of self-harm

Among patients with a history of self-harm, the most frequent antipsychotic choice on admission was risperidone (34.1%, Table 2), followed by olanzapine (29.3%) and aripiprazole (22.8%). Those with a history of self-harm (4.1%) were more likely to have used clozapine than those without (0.8%, exact *p* = 0.009). Antipsychotic choices did not differ between participants with and without a history of self-harm. Only of 2/79 patients with a history of suicidal behavior and of 1/57 patients with a history of suicide attempts used clozapine. Other associations between antipsychotic use and other types of self-harm were not statistically significant. None of patients were administered long-acting injectable (LAI) antipsychotics.

**Table 2:** Antipsychotic use on admission by different types of self-harm among inpatients with early-stage schizophrenia (*N* = 1057)

Variables	Total	Without self-harm	Self-harm	Suicidal behavior	Suicide attempt	Interrupted attempt	Aborted attempt	NSSI	SHPS	Preparatory acts
	*n* = 1057	*n* = 927	*n* = 130	*n* = 79	*n* = 57	*n* = 19	*n* = 8	*n* = 50	*n* = 25	*n* = 3
Amisulpride	33 (3.2%)	28 (3.1%)	5 (4.1%)	4 (5.3%)	2 (3.6%)	2 (11.1%)	0 (0.0%)	0 (0.0%)	2 (4.3%)	0 (0.0%)
Aripiprazole	208 (20.4%)	180 (20.1%)	28 (22.8%)	15 (19.7%)	13 (23.6%)	2 (11.1%)	0 (0.0%)	2 (66.7%)	14 (30.4%)	3 (13.0%)
Blonanserin	3 (0.3%)	2 (0.2%)	1 (0.8%)	0 (0.0%)	0 (0.0%)	0 (0.0%)	0 (0.0%)	0 (0.0%)	1 (2.2%)	1 (4.3%)
Chlorpromazine	1 (0.1%)	1 (0.1%)	0 (0.0%)	0 (0.0%)	0 (0.0%)	0 (0.0%)	0 (0.0%)	0 (0.0%)	0 (0.0%)	0 (0.0%)
Clozapine	12 (1.2%)	7 (0.8%)	5 (4.1%)	2 (2.6%)	1 (1.8%)	1 (5.6%)	0 (0.0%)	0 (0.0%)	3 (6.5%)	0 (0.0%)
Haloperidol	4 (0.4%)	4 (0.4%)	0 (0.0%)	0 (0.0%)	0 (0.0%)	0 (0.0%)	0 (0.0%)	0 (0.0%)	0 (0.0%)	0 (0.0%)
Olanzapine	267 (26.2%)	231 (25.8%)	36 (29.3%)	20 (26.3%)	14 (25.5%)	5 (27.8%)	3 (37.5%)	1 (33.3%)	16 (34.8%)	9 (39.1%)
Paliperidone	35 (3.4%)	32 (3.6%)	3 (2.4%)	2 (2.6%)	2 (3.6%)	0 (0.0%)	0 (0.0%)	0 (0.0%)	1 (2.2%)	1 (4.3%)
Quetiapine	75 (7.4%)	66 (7.4%)	9 (7.3%)	7 (9.2%)	4 (7.3%)	2 (11.1%)	3 (37.5%)	0 (0.0%)	1 (2.2%)	1 (4.3%)
Risperidone	408 (40.0%)	366 (40.8%)	42 (34.1%)	29 (38.2%)	22 (40.0%)	6 (33.3%)	2 (25.0%)	0 (0.0%)	12 (26.1%)	10 (43.5%)
Sulpiride	2 (0.2%)	2 (0.2%)	0 (0.0%)	0 (0.0%)	0 (0.0%)	0 (0.0%)	0 (0.0%)	0 (0.0%)	0 (0.0%)	0 (0.0%)
Ziprasidone	24 (2.4%)	19 (2.1%)	5 (4.1%)	4 (5.3%)	3 (5.5%)	1 (5.6%)	0 (0.0%)	0 (0.0%)	1 (2.2%)	0 (0.0%)

*Abbreviations*: NSSI, non-suicidal self-injury; SHPS, self-harm due to psychotic symptom; N, number.

### Switching to clozapine during hospitalization

**Fig. 2:**
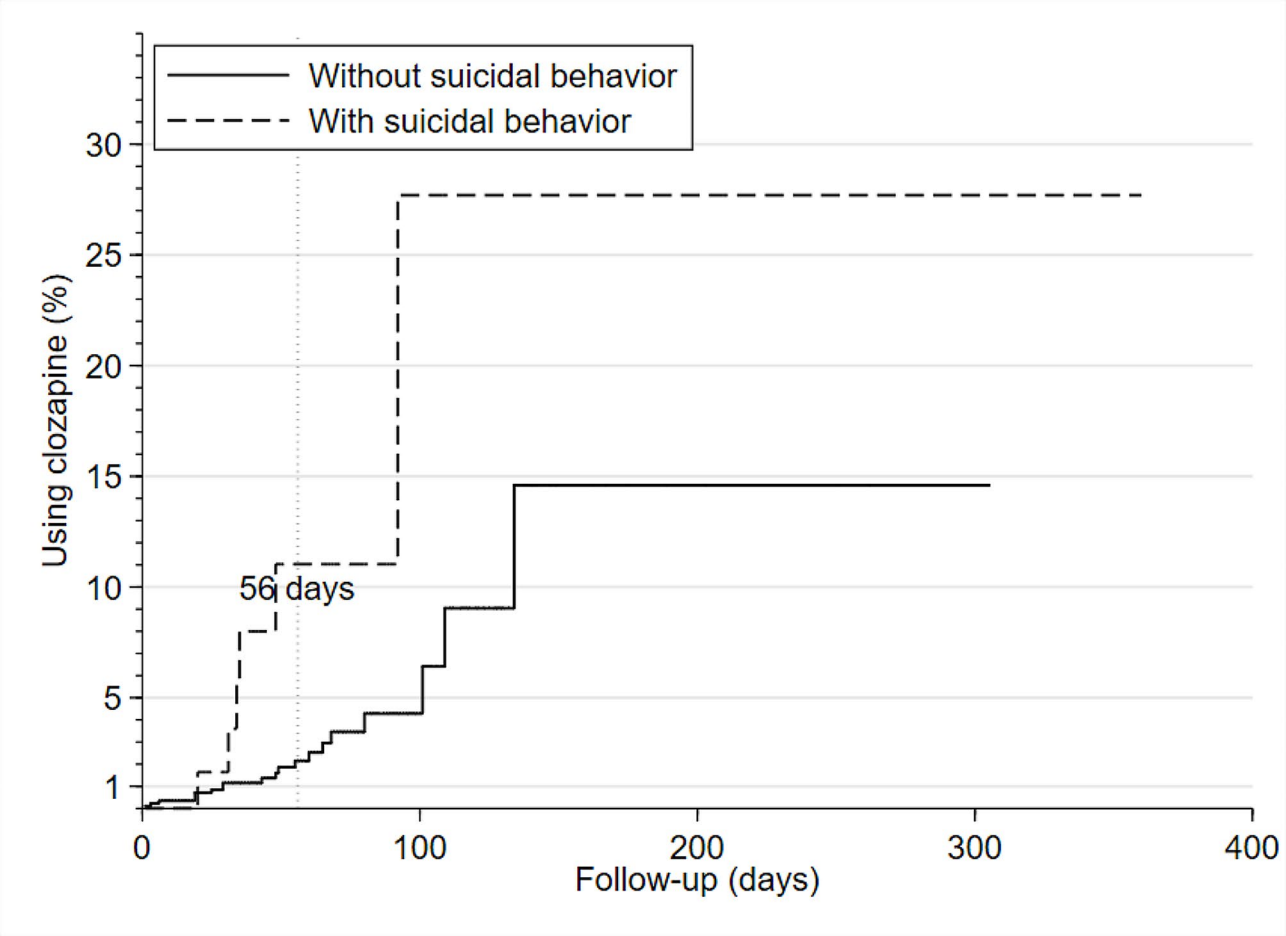
Cumulative hazard of switching to clozapine during hospitalization by the baseline history of suicidal behavior (without/with)

Of the original 1057 participants (Figs. 1), 2 were discharged from the hospital on the day of admission, 35 did not have information for antipsychotic use, 12 used clozapine on admission, 35 had a low WBC or ANC from the first blood test after admission, none had a record of neutropenia, and 6 had a diagnosis of leukopenia or thrombocytopenia. Thus, the analysis cohort comprised 967 inpatients.

The median follow-up duration was 37 (IQR = 44, Range: 1–360) days. The follow-up period did not differ between patients with and without a lifetime history of suicidal behavior (Median [IQR]: 42[64] vs. 44[36]; *z* = 0.29, *p* = 0.775). The total analysis time at risk and under observation was 48,433 person-days. No patient died during the follow-up period.

During hospitalization, 26 (2.7%) patients started using clozapine. Among these 26 patients, 7 used one antipsychotic from admission to initiating clozapine, 11 used two different antipsychotics, 7 used three antipsychotics, and 1 used four antipsychotics (Supplementary Table S2). No difference was found in the number of antipsychotics administered before clozapine uses between those with and without a history of suicidal behavior (exact *p* = 1.00). The proportion of patients who initiated clozapine was 6/73 for those with suicidal behavior, which was higher than that for those without (2.3%, exact *p* = 0.010). During the 56-day follow-up, 5 of 73 individuals with suicidal behavior switched to clozapine compared with 13 of 874 among those without a history of suicidal behavior (6.8% vs. 1.5%, exact *p* = 0.008).

The Cox PH regression showed that a history of suicidal behavior was associated with a higher possibility of switching to clozapine during hospitalization (Hazard ratio [HR] = 3.60, *p* = 0.006). After adjusting for age group, sex, and minimal treatment (yes/no), the Cox PH regression demonstrated that those with a history of suicidal behavior had a higher possibility of a shorter time to the start of using clozapine during hospitalization (adjusted HR = 4.18, 95% CI = 1.62–10.78, *p* = 0.003, Table 3; Fig. 2) or within 56 days after admission (adjusted HR = 6.09, 95% CI = 2.08–17.83, *p* = 0.001).

**Table 3:** Cox proportional hazards regression of baseline suicidal-behavior history on switching to clozapine during hospitalization or 56-day follow up (*N* = 967)

	Switching to clozapine during hospitalization				Switching to clozapine within 56-day follow up			
Variables		*95% CI*				*95%*	*CI*	
	*AHR*	*LL*	*UL*	*p*	*AHR*	*LL*	*UL*	*p*
Life-time suicidal behavior								
No	1(reference)				1			
Yes	**4.18**	**1.62**	**10.78**	**0.003**	**6.09**	**2.08**	**17.83**	**0.001**
Sex								
Male	1				1			
Female	0.41	0.18	0.93	0.032	0.50	0.19	1.31	0.159
Age groups			.	.				
10–24	1				1			
25–39	0.53	0.21	1.30	0.163	0.27	0.07	0.95	0.041
40–59	0.54	0.17	1.67	0.283	0.55	0.15	2.02	0.371
Treatment history								
Treated	1				1			
Minimal treated	0.88	0.39	1.96	0.749	0.79	0.30	2.06	0.630

*Abbreviations*: *AHR*; adjusted Hazard Ratio; 95% *CI*, 95% confidence intervals; *p*, *p*-values; LL, lower limit of the confidence interval; *UL*, upper limit of the confidence interval

Note: Results statistically significant at *p* < 0.05 level are in bold.

## Discussion

This real-world study yielded three important findings regarding individuals with schizophrenia in China. First, more than one in ten patients had reported a history of self-harm on admission. Second, a lifetime history of self-harm was associated with a higher possibility of clozapine uses on admission. Third, a lifetime history of suicidal behavior increased the possibility of initiating clozapine treatment during hospitalization or within 56 days after admission.

### Antipsychotic use

In our study, four-fifths of the patients used risperidone, olanzapine, and aripiprazole on admission. Clozapine use was uncommon (1.2% on admission and 2.7% started during hospitalization) for the treatment of early-stage schizophrenia at our sample site. Another study on drug-naïve first-episode schizophrenia spectrum disorders in a psychiatric hospital in Hunan, China reported a higher clozapine use rate (7.5%), which was 4.5% in tertiary hospitals and 11% in non-tertiary hospitals [29]. Their clozapine use rate in tertiary hospitals was similar in our study (also a tertiary hospital). This explanation may be because psychiatrists in high-level hospitals adherence more to China’s national clinical guidelines [29]. In China, clozapine is indicated only for treatment-resistant schizophrenia [14].

### Antipsychotic use by the history of suicidal behavior

Our study found that psychiatrists preferred to clozapine or changed prescription to it sooner when an admitted patient had a lifetime history of suicidal behavior. The first reason may be the psychiatrists acknowledged that, in the United States, clozapine is the only antipsychotic with an indication for treating suicide risk [12]. International best practices influence clinical decision making. The second reason may be a history of suicidal behavior increased their clinical prediction of the risk of treatment-resistant schizophrenia, an indication for clozapine use. Considering the small number of patients using clozapine, there may be potential chance reasons for the increased likelihood of switching to clozapine earlier among those with a history of suicidal behavior. Future research should test these findings using a larger sample size.

None of the patient used LAI antipsychotics, although LAI antipsychotics (risperidone, paliperidone, and perphenazine) were available at research site. Our sample consisted of inpatients with a short duration of schizophrenia. Then, the evidence of LAI antipsychotics and subsequent suicides has been rare [30].

### Implications

Suicide prevention is a challenge in the treatment of schizophrenia in psychiatric hospitals. A previous study reported that the majority (64/79) of suicidal events in a Chinese psychiatric hospital occurred from those with a diagnosis of schizophrenia [31]. Our study provides clinicians with information on the prevalence of self-harm among individuals with early-stage schizophrenia, suggesting the need to prevent their risk of self-harm and suicide. The findings about antipsychotic use upon admission and during hospitalization suggest the use of clozapine is not as popular as risperidone, olanzapine, and aripiprazole. The guidelines of treatment for schizophrenia should emphasize the underutilization among patients with a history of self-harm. In fact, clozapine is underutilized in many countries [32], although research suggests that clozapine reduces self-harm [7–9] and mortality compared with other antipsychotics [10, 33]. Underutilization may become a barrier to recovery from schizophrenia and high suicide risk.

In addition, our results showed that clozapine is utilized sooner for those patients with a history of suicidal behavior, who may benefit in reducing suicide risk from clozapine use. This survey on clinical practice can help psychiatrists know what other psychiatrists did when treating schizophrenia patients and not to be too cautious about clozapine. Then, clinicians should be trained to become more familiar with managing the risks and side effects of clozapine and its efficacy in preventing suicide and increasing life expectancy [34]. This training could change the situation of “clozaphobia” and improve the prescribing of clozapine for those really need it. In clinical practice, psychiatrists can choose tools such as multidimensional clozapine side-effect management [33] and therapeutic drug monitoring are essential tools, to increase clozapine safety. Reducing the clozapine dose is necessary for patients with reduced CYP2D6 (cytochrome P450 2D6; CYP2D6 poor metabolizers) activity. It is also important to regularly monitor the ANC on a regular basis for at least 2 years [35]. Comprehensive care for other side effects, such as cardiovascular risk, metabolic issues, and clozapine-induced constipation, would also optimize the treatment. One obstacle is implementing the implementation of evidence-based strategies.

### Strength

This real-world study used EMR’s prescription data to minimize information bias regarding antipsychotic use. Moreover, before analyzing the clozapine prescriptions in the cohort, we used blood test data and diagnoses of leukopenia or thrombocytopenia from the EMR to exclude clozapine contraindications. We also reported the short-term prescription switch that will help other practitioners choose an earlier use of clozapine.

### Limitations

This study has several limitations. First, the findings may be confounded by unmeasured factors, such as psychotic symptom severity, personal choice, previous antipsychotic use, antidepressants, ECT therapy, and self-harm during hospitalizations. Moreover, missing information on antipsychotic use before hospitalization and antipsychotic combination practice made it difficult to evaluate whether patients received a full course of treatment. Thus, we could not determine whether the patients who received clozapine in this study aligned with or violated the label.

Second, recall bias, stigma, and patient’ incapability may lead to reduced self-harm disclosure. The absence of a description of self-harm or suicidal behavior in medical records does not always indicate the absence of such behaviors. However, because our data were obtained from the inpatient department, the history of self-harm behavior was checked repeatedly by the entire clinical team, including the consultant psychiatrist, attending doctor, resident doctor, and nurses. Moreover, the bias in suicidal reporting may not have influenced our finding of an association between self-reported history of self-harm and psychiatrists’ decisions on antipsychotics. If the clinical team did not collect information on the history of suicide, psychiatrists would not consider using clozapine to reduce suicide risk.

Third, the data were collected from only one site, limiting the generalizability of the findings. However, we did not exclude patients who had received short-term schizophrenia treatment at other hospitals. Prescriptions at admission also reflected the practice in the previous hospital.

Fourth, we only included inpatients. Hospital psychiatry care is common among individuals with acute-phase schizophrenia in China. Self-harm intention or behavior is an indication for hospitalization. This sample have overestimated the prevalence of self-harm and clozapine use among all patients with early-stage schizophrenia.

Fifth, the sample size of the Cox regression analysis was limited, and the confidence intervals were wide for some estimates.

Finally, we directly used the diagnosis of schizophrenia from the EMR. To help psychiatrists differentiate schizophrenia diagnosis from other psychoses caused by organic/toxic causes, every inpatient with psychosis symptoms in the research site was routinely required to undergo brain imaging, electroencephalography, and neuropsychological assessments. However, lumbar puncture or urinalysis was performed only when necessary.

## Conclusions

Approximately 7.5% of inpatients with early-stage schizophrenia had a lifetime history of suicidal behavior. A history of lifetime suicidal behavior was associated with a five-fold increase in the probability of starting clozapine prescriptions within a 56-day hospital stay. However, clozapine may be underutilized for patients with schizophrenia and suicide risk.

### Electronic supplementary material

Below is the link to the electronic supplementary material.

Supplementary Material 1

## Data Availability

Research data are not shared because of the regulations of the institute which holds the medical records.

## Abbreviations

ANC: absolute neutrophil count
CYP2D6: cytochrome P450 2D6
ECT: electric convulsive treatment
EMR: electronic medical records
LAI: long-acting injectable
NSSI: non-suicidal self-injury
SHPS: self-harm due to psychotic symptom
WBC: white blood cell count
