# Supplementary material for: History of suicidal behavior and clozapine prescribing among people with schizophrenia in China: a cohort study
Source: BMC Psychiatry. 2024 Jun 12;24:440. doi: 10.1186/s12888-024-05893-y (PMC11167794; doi:10.1186/s12888-024-05893-y)
Supplement: Supplementary file 1 — Supplementary Material 1 [file 12888_2024_5893_MOESM1_ESM.docx]

**Supplementary materials**

**Prescribing clozapine in China**

1. The label of Clozapine tablets

【Warnings】

Neutropenia

Because clozapine has a high risk of neutropenia, which is a serious life-threatening adverse reaction that can lead to severe infections and death. Therefore, clozapine should only be used in the treatment of treatment-resistant schizophrenia in patients who have failed to respond to a full course of treatment with at least two different antipsychotics.

…

【Indications】 This product is not only effective for positive symptoms of mental illness but also has a certain effect on negative symptoms. It is suitable for all subtypes of acute and chronic schizophrenia and has a good effect on hallucinations, delusions, and youth. It can also reduce affective symptoms associated with schizophrenia (e.g., depression, guilt, anxiety). For some patients who do not respond to traditional antipsychotic treatment or have poor efficacy, switching to this product may be effective. This product is also used to treat agitation, agitation, and delusions of mania or other psychotic disorders. Because it causes neutropenia, it is generally not suitable as the first choice of drug.

1. Zhao J, Shi S. Guideline on prevention and treatment of schizophrenia. second ed. Beijing: Chinese Medical Multimedia Press; 2015.

Page 66

(clozapine) Indications: (1) patients with **treatment-resistant schizophrenia**; (2) patients with schizophrenia and severe delayed dyskinesia; (3) patients with schizophrenia prone to extrapyramidal adverse effects; (4) schizoaffective disorders, refractory mania, and severe psychotic depression; (5) psychotic symptoms secondary to antiparkinsonian medications, and low-dose clozapine (25-75 mg/d) is effective; (6) **patients with schizophrenia with severe suicidality**; (7) other refractory psychiatric illnesses: refractory patients with pervasive developmental disorder, autism, or obsessive-compulsive disorder.

**Table S1** Duration from self-harm to hospitalization (N = 52)

| Duration from self-harm to hospitalization (days) | Numbers | % |
| --- | --- | --- |
| 0–30 | 23 | 44.2 |
| 31–180 | 7 | 13.5 |
| 181–365 | 10 | 19.2 |
| 366–2045 | 12 | 23.1 |

Table S2 Antipsychotics prescribing for those used clozapine.

| id | Life-time suicide behavior | Days from hospitalization to initiating clozapine | Number of previous antipsychotics before clozapine | Drug sequence |
| --- | --- | --- | --- | --- |
| 1 | 0 | 1 | 1 | RISP CLOZ |
| 2 | 0 | 109 | 3 | RISP PALI OLAN CLOZ |
| 3 | 1 | 35 | 2 | OLAN ARIP CLOZ |
| 4 | 1 | 20 | 1 | OLAN CLOZ |
| 5 | 0 | 48 | 3 | PALI OLAN SULP CLOZ |
| 6 | 1 | 34 | 1 | RISP CLOZ QUET |
| 7 | 1 | 48 | 3 | RISP ARIP OLAN CLOZ |
| 8 | 0 | 49 | 2 | RIRSP OLAN CLOZ |
| 9 | 0 | 134 | 2 | RIRSP OLAN CLOZ |
| 10 | 0 | 101 | 3 | OLAN ZIPR PALI CLOZ |
| 11 | 0 | 3 | 1 | OLAN CLOZ |
| 12 | 0 | 68 | 3 | AMIS RISP OLAN CLOZ |
| 13 | 0 | 19 | 2 | RISP SULP CLOZ |
| 14 | 0 | 55 | 2 | ARIP RISP CLOZ |
| 15 | 0 | 43 | 4 | ARIP RISP OLAN SULP CLOZ |
| 16 | 0 | 6 | 1 | ARIP CLOZ RISP |
| 17 | 0 | 60 | 2 | RISP QUET CLOZ |
| 18 | 0 | 25 | 1 | OLAN CLOZ |
| 19 | 0 | 29 | 3 | OLAN RISP AMIS CLOZ |
| 20 | 0 | 29 | 1 | OLAN CLOZ |
| 21 | 0 | 19 | 2 | OLAN RISP CLOZ |
| 22 | 0 | 65 | 2 | OLAN ARIP CLOZ |
| 23 | 0 | 80 | 2 | OLAN RISP CLOZ ARIP |
| 24 | 1 | 92 | 3 | ARIP QUET HALO CLOZ |
| 25 | 0 | 19 | 2 | RISP OLAN CLOZ ARIP |
| 26 | 1 | 31 | 2 | ZIPR HALO CLOZ |

Abbreviations:

ARIP: Aripiprazole AMIS: Amisulpride CLOZ: Clozapine

HALO: Haloperidol OLAN: Olanzapine QUET: Quetiapine

RISP: Risperidone SULP: Sulpiride ZIPR: Ziprasidone
